# Hard to Please

**Published:** 1877-04

**Authors:** 


HARD TO PLEASE.—We have seceived a communication from a city subscriber who complains of our club-list. Says he sees nothing in it that exactly suits his fancy. Wants to know whether we can club with chicken salad, terrapin soup, or something substantial, of the nature indicated. It is plain to be seen that our friend has had too much clubbing already, and that the recollections of the last gastronomic club meeting still lingers in his mind, creating a sympathetic desire in his stomach. What a club list that would make to be sure, and how it would tickle the palates of a jolly lot of Clubites we wot of. Barry Gray's *Table*, a journal devoted to the wild and varied fancies of the gastronomer, expired, alas too soon, else might we have placed it upon our clubbing list as a gentle reminder of what might be accomplished in the art to which they are most devoted.

